# Multidimensional pooled shRNA screens in human THP-1 cells identify candidate modulators of macrophage polarization

**DOI:** 10.1371/journal.pone.0183679

**Published:** 2017-08-24

**Authors:** Ewa Surdziel, Ieuan Clay, Florian Nigsch, Anke Thiemeyer, Cyril Allard, Gregory Hoffman, John S. Reece-Hoyes, Tanushree Phadke, Romain Gambert, Caroline Gubser Keller, Marie-Gabrielle Ludwig, Birgit Baumgarten, Mathias Frederiksen, Dirk Schübeler, Klaus Seuwen, Tewis Bouwmeester, Barna D. Fodor

**Affiliations:** 1 Novartis Institutes for Biomedical Research, Basel, Switzerland; 2 Novartis Institutes for Biomedical Research, Cambridge, United States of America; 3 Friedrich Miescher Institute for BioMedical Research, Basel, Switzerland; University of Michigan Health System, UNITED STATES

## Abstract

Macrophages are key cell types of the innate immune system regulating host defense, inflammation, tissue homeostasis and cancer. Within this functional spectrum diverse and often opposing phenotypes are displayed which are dictated by environmental clues and depend on highly plastic transcriptional programs. Among these the ‘classical’ (M1) and ‘alternative’ (M2) macrophage polarization phenotypes are the best characterized. Understanding macrophage polarization in humans may reveal novel therapeutic intervention possibilities for chronic inflammation, wound healing and cancer. Systematic loss of function screening in human primary macrophages is limited due to lack of robust gene delivery methods and limited sample availability. To overcome these hurdles we developed cell-autonomous assays using the THP-1 cell line allowing genetic screens for human macrophage phenotypes. We screened 648 chromatin and signaling regulators with a pooled shRNA library for M1 and M2 polarization modulators. Validation experiments confirmed the primary screening results and identified OGT (O-linked N-acetylglucosamine (GlcNAc) transferase) as a novel mediator of M2 polarization in human macrophages. Our approach offers a possible avenue to utilize comprehensive genetic tools to identify novel candidate genes regulating macrophage polarization in humans.

## Introduction

Macrophages are the most important line of innate immune defense found in all tissues where they contribute to immune responses, wound healing and regulation of inflammation [1–4]. These seemingly opposing functions are attributed to the high degree of macrophage transcriptional plasticity as they can change their profiles depending on the microenvironment [5]. Although distinct subpopulations of macrophages with unique functional abilities have been described, it is believed that these are not so sharply demarcated and rather represent a spectrum of activated phenotypes [6–9]. Among the several activation subtypes the best characterized are the ‘classically activated’ (also termed M1) and ‘alternatively activated’ (also termed M2) macrophages. M1 macrophages mediate host defense against bacteria, protozoa and viruses and are induced by IFNγ and microbial products such as Toll-like receptor (TLR) ligands. Alternative macrophages exhibit immune suppressive function and can be activated e.g. by T helper (Th)2 cytokines (IL4 and IL13), immune complexes, glucocorticoids, TGFβ and IL10 [10,11].

Macrophage subtype specialization is regulated by several transcriptional factors and chromatin regulators [12]. Furthermore, these factors may impose epigenetic modifications that persist once the original environmental stimulus has ceased and thus provide a mechanism for extending the transient signals into a sustained cellular response lasting some hours or even days [12]. To date, several epigenetic factors such as Jmjd3, Hdac and BET family members have been shown to affect M1 / M2 polarization in mouse *in vivo* and *ex vivo* models [13–19].

Previous approaches to identify and / or validate regulators of macrophage biology employed siRNA technology [20–22]. However, the limitation of this approach is the transient nature of the knockdown effect which may not reveal long term epigenetic modulation. With the advent of pooled screening approaches using shRNA- or more recently clustered regularly interspaced short palindromic repeats (CRISPR) associated nuclease Cas9-libraries, long-term large-scale functional identification of genes in various mammalian models became more accessible [23–29]. Recently, this strategy has been used in a rodent model providing important insights into the regulation of Tlr4 signaling in DCs [25,30]. Nevertheless, translating this knowledge to humans is limited due to significant differences between mice and humans in immune system development, activation, and response [31–34]. With respect to monocytes and macrophages, direct use of human primary cells for genetic screens is hindered by the poor efficiency of their genetic manipulation and limited amounts available for large-scale *ex vivo* studies. To overcome these limitations we developed a cell-autonomous, physiologically relevant assay using THP-1 cells that is compatible with lentivirus based pooled shRNA screening. Using a library against 648 genes mainly covering transcriptional and chromatin regulators we identified several candidates potentially relevant for macrophage polarization in humans. We further validated OGT with small molecule and CRISPR-Cas9 mediated gene disruption as a novel regulator of M2 polarization. In summary, we outline here a streamlined experimental strategy that employs robust genetic tools to identify candidate modulators of human macrophage polarization.

## Materials and methods

### THP-1 cell culture, macrophage differentiation and polarization

Human leukemia monocytic THP-1 cells were purchased from the Leibniz Institute DSMZ-German Collection of Microorganisms and Cell Cultures (Cat. Nr. ACC1, Braunschweig, Germany). Parental and transgenic THP-1 cells were maintained in RPMI 1640 (Gibco) supplemented with 10% fetal calf serum (FCS) and 2mM L-glutamine. THP-1 monocytes (Mo) were differentiated into resting macrophages (M0) using 100 nM phorbol 12-myristate 13-acetate (PMA, Sigma-Aldrich) for 72 h followed by 24 h in PMA-free medium (PMA-resting, PMAr). For M1 / M2 polarization M0 macrophages were further cultured in M1-polarization medium containing 100 ng / ml LPS (Sigma-Aldrich) and 20 ng / ml IFNγ (R&D) for 24 h or in M2-polarization medium containing 25 ng / ml IL4 (R&D) and 25 ng / ml IL13 (eBioscience) for 48 h starting on the third day of PMA treatment. Cells were cultured at 37°C in a humidified 5% CO_2_ air atmosphere. OSMI-1, LMK-235 and PFI-3 were purchased from Sigma-Aldrich.

### Isolation of primary human monocytes, macrophage generation and polarization

Human monocytes (purity > 90% CD14+) were obtained from healthy donors buffy coats by 2-step gradient centrifugation followed by an additional step using the EasySep Human CD14 Positive Selection kit (Stemcell Technologies). Isolated human monocytes were cultured for 3 days in IMDM, 10% AB human serum, 1x NEAA, 2 mM Glutamax, 1 mM Na-Pyruvate, 4 μg / ml human insulin, 1% Pen/Strep (all from Invitrogen) supplemented with 5 ng / ml M-CSF (R&D). M1- or M2-polarized macrophages were obtained by further addition of 10 ng / ml IFNγ or 20 ng / ml IL4 (both from R&D), respectively. Cells were cultured at 37°C in a humidified 5% CO_2_ air atmosphere. Blood samples for the buffy coat preparations were obtained from the Swiss Red Cross which were collected using written informed consent. The use of samples was approved by the Ethikkommission Nordwest-und Zentralschweiz.

### Antibodies and cell viability reagents

The following monoclonal antibodies were used for flow cytometry and immunofluorescence: anti-CD11b-APC (ICRF44), anti-CD11c-PerCP / Cy5.5 (3.9), anti-CD38-AlexaFluor488 (HIT2), anti-CD38-V450 (HIT2), anti-CD209-AlexaFluor647 (9E9A8) along with the corresponding isotype controls (all from BioLegend). Anti-FLAG-FITC (M2) and IgG1-FITC isotype control (MOPC 21) (both from Sigma Aldrich). Cell viability was assessed by Trypan Blue count (Invitrogen) and Zombie Violet staining (BioLegend). For western blot analysis anti-OGT (ab96718, Abcam) and anti-β-ACTIN (8H10D10, Cell Signaling Technology) were used.

### Protein isolation and western blot analysis

THP-1 cells were lysed in RIPA buffer (Cell Signaling Technology) and proteins were extracted by repeated freeze-thaw cycles followed by Benzonase (Novagen) treatment. 30 μg lysate was loaded per lane and the proteins were separated using Novex NuPAGE SDS-PAGE gel system transferred to Novex Invitrolon PVDF membranes and subjected to immunoblotting. Luminescence was acquired with the Image Reader LAS-4000 (GE Healthcare Life Sciences).

### Flow cytometry

Flow cytometry data was acquired using a CyAn ADP Analyzer (Beckman Coulter) and BD FACSAria II (BD) flow cytometers. FcRs-unspecific binding was blocked by incubating cells in 100 μg / ml IgG from human serum (Sigma-Aldrich). Cells were suspended in Cell Staining Buffer (BioLegend) and incubated with monoclonal antibodies. For the flow cytometric analysis of Cas9-FLAG expression, THP-1 cells were fixed and permeabilized with the BD Cytofix / Cytoperm (BD Biosciences) according to the manufacturer protocol and stained with the anti-FLAG antibody. Fluorescence was compared to the corresponding isotype-stained controls. All data were analyzed using the FlowJo software (Tree Star Inc.).

### Cytokine and chemokine release profiling

THP-1 cells were polarized as described in the preceding paragraph. Supernatant was collected from three independent experiments, pooled and subjected to the Proteome Profiler (ARY005, R&D Systems) according to the manufacturer’s protocol. Luminescence was acquired with the Image Reader LAS-4000 (GE Healthcare Life Sciences) and densitometry done using the ImageJ software.

### Microarray transcriptional profiling of THP-1 and human primary macrophages

THP-1 monocytes and human primary macrophages were cultured and differentiated as described in the preceding paragraphs. For the microarray profiling, 2 polarizing conditions were used in THP-1 model as follows: 1) 20 ng / ml IFNγ solely for 24 h, 2) 25 ng / ml IL4 solely for 48h. RNA was extracted using the RNeasy Mini Kit (Qiagen), *DNaseI* digested (Qiagen) and RNA quality was evaluated using the Agilent RNA 6000 Nano Kit and 2100 Bioanalzer (Agilent Technologies). Total RNA (8 μl at 50 ng / μl) were supplied to the Genome Array Lab for reverse transcription, labeling and hybridization using an Affimetrix GeneChip Human Genome U133 Plus 2.0 Array (HG-U133_Plus_2). The transcriptional profiles were evaluated in three independent cell preparations, each derived from a different single donor (primary monocytes) or from a separate stimulation experiments (THP-1). Microarray data were uploaded to ArrayExpress for the primary cells and the THP-1 cells with accession numbers E-MTAB-5913 and E-MTAB-5917, respectively.

### Microarray data analysis

Fold changes (FCs) between samples were calculated using the mean value of three independent hybridizations and adjusted p-values. Quality control was performed in R using the Bioconductor package array QualityMetrics (http://www.bioconductor.org). RMA gene expression levels (robust multi-array average) were calculated using the Bioconductor packages Affycoretools and Affyio. Lists of differentially expressed probe sets were produced with FC and t-test p-value calculation using the Bioconductor packages Limma, reshape and Plyr, using a factorial design approach to extract the relevant comparisons. For each comparison, differentially expressed genes were defined as those exhibiting an absolute FC ≥ 1.5 and p-value < 0.05. Annotation data for probes was obtained using the Bioconductor packages AnnotationDbi and hgu133plus2.db.

### Pooled lentiviral shRNA library

A previously described, custom 12,998 element shRNA library focused on epigenetic and signaling regulators targeting 648 genes was used for screening [29]. On average each gene was covered by 20 unique shRNAs and the oligo corresponding to each shRNA was synthesized with a unique 18 nucleotide barcode (Cellecta) for measuring representation by NGS.

### Individual shRNA and sgRNA constructs

Individual shRNA constructs were cloned into pRSI16 lentiviral plasmid (Cellecta) as described previously [23] with unique sequences corresponding to the target: 5’-GACGCAACCGAACTTTGCAGT-3’ (shRNA-OGT_v1), 5’-CCAAACTTTCTGGATGCTTAT-3’ (shRNA-OGT_v2), 5’-TGTTGCAGATGGGTGATATAT-3’ (shRNA-OGT_v3) and 5’-CAAATCACAGAATCGTCGTAT-3’ (shRNA-Luc). Individual sgRNA constructs were cloned into pNGx_LV_g003 [27] with unique sequences corresponding to the target: 5’-GGAAAGAGGGCAGTTGCAGG-3’ (sgRNA-OGT_v1), 5’-GGGTCGCTTGGAAGAAGCCA-3’ (sgRNA-OGT_v2), 5’-TAATGGGCACACCACAGGGA-3’ (sgRNA-OGT_v3) and 5’- GTAGCGAACGTGTCCGGCGT -3’ (sgRNA-CTRL).

### Lentivirus production, cell transduction and pooled screening

Viral packaging was performed as described before [23]. To perform large-scale transduction, 8.5 x 10^7^ of THP-1 cells were transduced at MOI of 0.3 to ensure single shRNA integration per cell. The optimal puromycin (Invitrogen) dose required to achieve > 95% cell killing in 72h was determined by measuring cell viability with a Cell Titer Glo assay (Promega) for a dose response ranging from 0 to 4 μg puromycin. The volume of virus required to give an MOI of 0.3 was determined using a 10 point dose response ranging from 0 to 300 μL of viral supernatant in the presence of 5 μg / mL polybrene (Millipore). Infectivity was determined as the % RFP+ cells as measured by flow cytometry. A spin infection was performed in the presence of 5 μg / ml polybrene by centrifugation at 2000 rpm for 1 h at 32°C. 48h post-transduction cells were washed with PBS (Gibco) and 1 μg / ml puromycin was added. 72 h following puromycin addition (5 days post-transduction), an aliquot of cells was used to measure transduction efficiency determined by measuring the % RFP+ cells and was typically > 90%. Simultaneously, 2 x 10^7^ cells serving as an input sample were collected and stored at -80°C for genomic DNA (gDNA) isolation and NGS. The rest of the cells were subjected to the following treatments: no treatment, M1 polarization or M2 polarization. After 4 days of incubation (9 days post-transduction) cells were collected, stained with monoclonal antibodies and subjected to the preparative FACS of RFP+ cells expressing a desired phenotype. Sorted cell fractions (positive and negative) were collected and subjected to the gDNA isolation and NGS along with the input sample. To elucidate essential genes THP-1 cells stably expressing the epigenetic library were left untreated (“viable Mo”) and subjected to the NGS along with the input sample (S1 Fig). Comparing “viable Mo” and input sample identified essential genes. Sorting was performed with two biological replicates (R^2^ ≥ 0.97, see S2 Fig) except for the M1 negative fraction for which one biological replica was used. Screens were run in biological duplicates. Lentiviral particle containing individual shRNAs or sgRNAs were produced in 6-well plates by adjusting the above protocol. For single shRNA-mediated knockdown, THP-1 cells were transduced and analyzed following the pooled shRNA screening protocol. For generating Cas9 expressing cells lentiviruses packaged with pLenti6_CMV_3xFLAG_nls-SPyCas9_nls_T2a_Blast [28] were transduced into THP-1 cells and selected with 5 μg / ml Blasticidin for stable integration. For single CRISPR / sgRNA-mediated knockdown, THP-1 cells stably expressing Cas9 were transduced according to the pooled shRNA screening protocol, but analyzed 11 days post-transduction.

### Purification of genomic DNA, library production and next generation sequencing

Cells in a range from 0.7 up to 20 million cells were resuspended in PBS according to the DNeasy protocol (Qiagen). Resuspension was then aliquoted, treated with ProteinaseK, RNaseA and Buffer AL and incubated for lysis and processed for gDNA isolation. The final DNA concentration was assayed using Picogreen reagent.

For NGS library generation, the barcodes were amplified in 8 independent 50 μL PCR reactions using 1 μg of gDNA per reaction with Titanium Taq and Primers #3323 (PEFwdGEX), #3324 (PECellectaA), and #3197–3223 (one of 27 indexing oligos) for 28 cycles. The product was analyzed by agarose gel electrophoresis to check for the expected ~120bp product and purified using the Agencourt. AMPure XP PCR cleanup kit (Beckman Coulter) and the amount of purified product quantified using a Picogreen DNA concentration assay. Barcode representation of each barcode in the 12,998 element shRNA library (Cellecta) was measured by NGS on an Illumina GA2X system. For good representation of each shRNA in the NGS data 12 million raw Illumina sequence reads were required per sample, which averages 2000 reads per shRNA. The deep coverage shRNA libraries used in this work enable high confidence hit calling at the gene level, rather than analysis of individual shRNAs in the data set.

### Pooled shRNA screen data analysis

Sequencing and data analysis were carried out as described previously [23,35]. Counts from each sample were normalized to 12 million reads. In brief, the number of reads observed for each barcode was divided by the number of reads for the corresponding barcode in the original plasmid pool to give the fold change in representation during the experiment. The fold changes for a set of reagents / barcodes targeting a gene were analyzed by means of the RSA (redundant siRNA activity) algorithm. A gene gets a better score, i.e. lower p-value, if the shRNAs against it are unusually distributed toward one of the extremities in a list of all the shRNAs sorted by fold change. Such sorted lists were generated from differential shRNA counts for (i) M1+ vs. input or vs. M1-, (ii) M2+ vs. input or vs. M2-, and (iii) M2+ vs. M1+ as described in the results section. p-values were calculated using a hypergeometric enrichment focusing only on shRNA depletion.

### Quantitative real-time reverse-transcription PCR

RNA was extracted using the RNeasy Mini Kit (Qiagen). cDNA synthesis (Applied Biosystems) was performed with *DNaseI*-digested RNA (Qiagen) and subjected to Human Cytokines & Chemokines RT2 Profiler PCR Array (Qiagen) following the manufacturer’s protocol. Real-time PCR was performed using ABI ViiA 7 cycler (Applied Biosystems). Data was analyzed following the manufacturer’s protocol and as described previously [36].

### High content imaging experiments and data analysis

For high content imaging experiments THP-1 cells were seeded into Microclear bottom 384-well plates (Greiner) at a density of 5000 cells / well and treated with 100 nM PMA for 24 h after which compounds were added at concentrations indicated in the corresponding section. On the third day PMA / compound medium was washed out and cells were cultured in compound-supplemented medium and stimulated with a combination of IL13 and IL4 at 25 ng / ml each for 48 h to induce M2 polarization. Afterwards, the FcRs-unspecific binding was blocked using 100 μg / ml IgG from human serum (Sigma-Aldrich), and M2 cells were stained with CD209-AlexaFluor647 (9E9A8). Subsequently, cells were fixed with FluoroFix buffer (Biolegend) and ultimately stained with Hoechst (Life Technologies). Images were obtained with the IN Cell Analyzer 2000 (GE Healthcare Life Sciences) and analyzed with Cell Profiler version 2.1.1 [37]. The median cell number analyzed in each well was 584. The signal was normalized to the corresponding isotype-stained DMSO controls and an intensity threshold was determined to bin CD209 positive and negative cells. The raw data obtained after image analysis is in S1 Table. Representative images shown were processed with FIJI [38] and the CD209-AlexaFluor647 staining was pseudo-colored yellow for better visualization.

## Results

### THP-1 myeloid cell line represents a model for human primary macrophages

shRNA based genetic screens require a suitable model available at large scale and compatible with lentiviral delivery of the library in order to reach the needed deep coverage. To screen for human macrophage polarization regulators we evaluated the well-established THP-1 model for these criteria [39,40].

**Fig 1:**
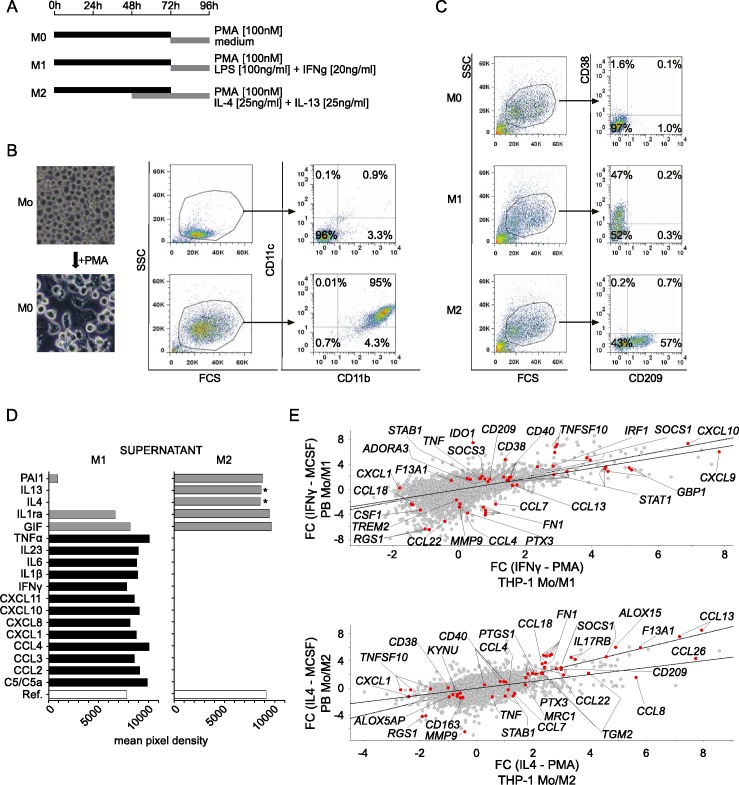
THP-1 monocyte to macrophage differentiation and M1 / M2 polarization. (A) Schematic representation of treatments to differentiate THP-1 monocytes (Mo) into macrophages (M0) or polarized macrophages (M1, M2). (B) Morphological and cell surface marker expression (CD11c, CD11b) changes observed by microscopic and flow cytometric analysis of PMA (phorbol 12-myrisate 13-acetate) differentiated M0. FCS: forward-scatter; SSC: side-scatter. (C) Resting CD38- / CD209- M0 macrophages (top panel) are polarized in CD38+ / CD209- M1 fraction upon LPS / IFNγ treatment (middle panel) and in CD38- / CD209+ M2 fraction upon IL4 / IL13 treatment (bottom panel). (D) Cytokine and chemokine release profiling of THP-1 M1 and M2 macrophages detecting M1 (black) and M2 (grey) specific soluble mediators, respectively. Supernatants were collected from three independent experiments and then pooled for the analysis. *Exogenously added to the M2 polarization medium. (E) Correlation analysis of transcriptional fold change (FC) during Mo to M1 (top panel) or M2 (bottom panel) polarization comparing THP-1 and human primary cells. For macrophage polarization MCSF (human primary cells) or PMA (THP-1) differentiated macrophages were treated with IFNγ (M1) or IL4 (M2). Whole genome microarray measurements were used from three independent experiments and each dot corresponds to a probe on the array. Highlighted genes are previously reported human markers relevant for the M1 or M2 polarization [32,31] (additional references listed in the main text). Highlighting was limited to genes which at least in one of the cellular models had at least one probe with 1 < or– 1 > FC. Dashed line is the y = x reference, solid line is fitted on the experimental data y = a + b * x (a = interception of y, b = slope).

Treatment of THP-1 monocytes with PMA ceased cell proliferation and induced differentiation into adherent CD11b and CD11c double positive resting macrophages referred to as M0 (Fig 1A and 1B). Furthermore, we established a polarization protocol yielding M1 and M2 cells induced by IFNγ / LPS or IL4 / IL13, respectively (Fig 1A and 1C). After testing a panel of cell surface markers we identified CD38 and CD209 (DC-SIGN) as unique for M1 or M2, respectively. Importantly, specificity of selected markers allowed subsequent fluorescence activated cell sorting (FACS) of M1 and M2 phenotypes.

One of the major roles of activated macrophages is the production of soluble mediators in response to environmental cues. To validate the biological relevance of the THP-1 model we measured cytokine and chemokine release profiles of THP-1 derived M1 and M2 macrophages (Fig 1D) [11,41]. We found that classically activated THP-1 macrophages release a wide spectrum of pro-inflammatory mediators such as IFNγ, TNFα, IL1β, IL6, IL23, CCL2, CCL3, CCL4, CXCL1, CXCL8, CXCL10, CXCL11 and the complement component C5 / C5a. On the contrary, in M2 polarized THP-1 supernatants we detected anti-inflammatory IL1RA, pro-fibrotic PAI1 (SERPINE1) produced by the cells, whereas release of M1-specific cytokines / chemokines was not detected. Since the M2 polarization medium contained exogenous IL4 and IL13 it was not possible to quantify intrinsic release of IL4 and IL13.

Next we compared the transcriptional changes between M1 or M2 polarized THP-1 and human primary macrophages (Fig 1E). We correlated fold change of gene expression from monocytes to M1 (IFNγ) or M2 (IL4) macrophage polarization in the corresponding THP-1 or human primary cells using whole genome microarray gene expression analysis. We observed comparable expression changes of well-established markers for both M1 (*CD38*, *CD40*, *CXCL9*, *CXCL10*, *GBP1*, *IDO1*, *IRF1*, *STAT1* and *TNFSF10*) and M2 (*ALOX15*, *CCL13*, *CCL18*, *CCL22*, *CCL26*, *CD209*, *F13A1*, *FN1*, *IL17RB*, *MRC1*, *PTGS1*, *SOCS1* and *TGM2*) when comparing the THP-1 macrophages to the corresponding human primary macrophages [32,31,41,42]. Extended expression data on other markers are shown in Fig 1E and the full data set can be accessed through ArrayExpress under accession numbers E-MTAB-5913 and E-MTAB-5917.

Taken together, our comprehensive data on gene expression signature and cytokine / chemokine release argue that the THP-1 model mimics human primary M1 / M2 macrophage subsets thus allowing identification of biologically relevant target genes.

### Multidimensional genetic screens in the human macrophage polarization model

Using the THP-1 model we performed two independent pooled genetic screens. We used a library of lentiviruses targeting 648 genes mainly involved in epigenetic regulation [29]. Each gene was targeted by 20 independent shRNAs each labeled with a unique barcode for subsequent identification by next generation sequencing (NGS).

**Fig 2:**
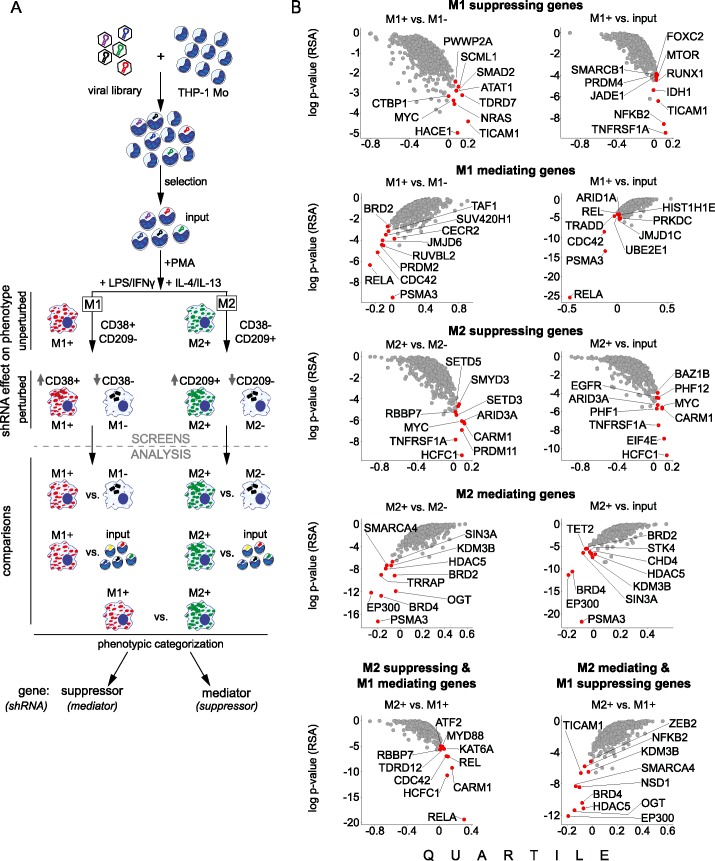
Pooled shRNA screening identifies candidate modulators of macrophage polarization in THP-1 cells. (A) Screening workflow for the phenotypic pooled shRNA screens to identify genes influencing macrophage differentiation and M1 / M2 polarization. Below, schematic representation of the analysis set up comprising different comparisons in two analysis modes (phenotype suppressor or mediator). (B) Analysis of screening results using the five comparisons and two analysis modes described in panel A. Top 10 hits are highlighted. Gene level scores were derived from shRNA reagents by calculating both the Redundant siRNA Activity (RSA) p-values as well as upper / lower quartiles. These two quantities describe significance and effect size, respectively, of a gene's knockdown by all reagents targeting this gene. Each sample was scaled to a total 12 million reads.

THP-1 monocytes were transduced at low MOI to ensure single shRNA integration per cell (Fig 2A). After puromycin selection, an aliquot was collected to determine library coverage, referred to hereafter as ‘input’ sample. The cells were then subjected to two different phenotypic screening paradigms from which additional fractions were sorted.

To decipher modifiers of M1 and M2 polarization we divided PMA-differentiated macrophages into two fractions and treated them with either IFNγ + LPS or with IL4 + IL13 to induce M1 or M2 subtypes, respectively. Next, M1+ (CD38+ / CD209-), M1- (CD38- / CD209-), as well as M2+ (CD38- / CD209+), and M2- (CD38- / CD209-) cells were sorted.

For NGS library generation, barcodes from the aforementioned conditions were amplified from genomic DNA extracted from cell pellets for each experimental condition. Subsequently, the count data obtained after sequencing were analyzed by means of the redundant siRNA activity (RSA) algorithm and quartile distribution allowing hit calling at the level of genes rather than individual shRNA hits (Fig 2B, S2 Table). In addition we checked reproducibility between biological replicas and identify genes affecting cell viability in our library with drop out screens (S1 Fig and S2 Fig).

### Identification of novel candidate genes regulating M1 / M2 macrophage polarization

Using the screening protocol described above we aimed to identify mediators and suppressors of macrophage polarization (Fig 2, Table 1 and S3 Table). To call hits regulating M1 polarization we compared M1+ (CD38+ / CD209-) to M1- (CD38- / CD209-), M2+ (CD38- / CD209+), or input (Fig 2B). We categorized the genes based on their putative normal function inferred from the knock down phenotype. Hence, genes for which the shRNAs were overrepresented in M1+ in any of the above comparisons were categorized as M1 suppressors. Genes for which shRNAs were underrepresented in the same comparisons were categorized as M1 mediators. Similarly, we compared M2+ (CD38- / CD209+) to M2- (CD38- / CD209-), M1+ (CD38+ / CD209-), or input to uncover M2 subtype suppressors (shRNAs overrepresented in M2+) and mediators (shRNAs underrepresented in M2+) (Fig 2B, Table 1 and S3 Table).

**Table 1:** Contingency table showing gene-centric representation of top 10 ranked hits from the M1 / M2 screens.

			**M2 modulation**							
			**number of screening comparisons in which a given gene was within the top 10 ranking hits as M2**							
			**suppressor**			** **	**mediator**			
			**3**	**2**	**1**		**1**	**2**	**3**	
**M1 modulation** **number of screening comparisons in which a given gene was within the top 10 ranking hits as M1**	**suppressor**	**3**	* *	* *	* *	* *	***TICAM1***	* *	* *	**3**
		**2**	* *	* *	* *	* *	***NFKB2***	* *	* *	**2**
		**1**	* *	***MYC*, *TNFRSF1A***	* *	*ATAT1*, *CTBP1*, *FOXC2*, *HACE1*, *IDH1*, *JADE1*, *MTOR*, *NRAS*, *PRDM4*, *PWWP2A*, *RUNX1*, *SCML1*, *SMAD2*, *SMARCB1*, *TDRD7*	*NSD1*, *ZEB2*	***OGT*, *SMARCA4***	***BRD4*, *EP300*, *HDAC5*, *KDM3B***	**1**
		** **	* *	***ARID3A***	*BAZ1B*, *EGFR*, *EIF4E*, *PHF1*, *PHF12*, *PRDM11*, *SETD3*, *SETD5*, *SMYD3*	* *	*CHD4*, *STK4*, *TET2*, *TRRAP*	***SIN3A***	* *	**0**
	**mediator**	**1**	***CARM1*, *HCFC1***	***RBBP7***	*ATF2*, *KAT6A*, MYD88*, *TDRD12*	*ARID1A*, *CECR2*, *HIST1H1E*, *JMJD1C*, *JMJD6*, *PRDM2*, *PRKDC*, *RUVBL2*, *SUV420H1*, *TAF1*, *TRADD*, *UBE2E1*	* *	BRD2*	* *	**1**
		**2**	* *	* *	REL*	* *	* *	***PSMA3***	* *	**2**
		**3**	* *	* *	*CDC42*, RELA*	* *	* *	* *	* *	**3**
			**3**	**2**	**1**	**0**	**1**	**2**	**3**	

Numbers indicate the number of screening comparisons in which a given gene was within the top 10 ranked hits.

“Mediator” and “suppressor” refer to the normal gene function inferred from the knock down phenotypes, respectively.

The central area demarcated by bold line contains genes which scored in only one comparison and were considered as lower confidence. The genes outside this area also highlighted by larger font size scored more than once, and were considered higher confidence.

*Known mediator of M1 polarization based on genetic data in mouse model.

Since RSA scores are not absolute but simply rank genes in a given experiment, we arbitrary took the top 10 ranking genes from each comparison as hits (Fig 2B and S3 Table). We argued that this cutoff, corresponding to 1.5% of the genes tested, should be stringent enough to enrich for true hits that can be later validated with orthogonal approaches. To further rank the top 10 hits, they were visualized in a contingency table based on the predicted function and the number of comparison they scored in (Table 1). Some genes fell into the same category in multiple comparisons (e.g. *BRD4*), while others scored in one comparison only (e.g. *ATAT1*) (Table 1). The latter were considered lower confidence hits. Furthermore, we observed that most of the candidate genes that modulated both M1 and M2 phenotypes suppressed one of the polarization states while mediated the other. For example *CARM1* scored as a suppressor of M2 and a mediator of M1. In contrast, some other genes that regulated both M1 and M2 were either suppressors of both polarization states, or mediators of both polarization states. For example *MYC* scored as a suppressor of M1 as well as suppressor of M2 polarization (Table 1). Our approach also identified genes which were previously shown by genetic manipulation of mice to regulate macrophage polarization such as *BRD2*, *RELA*, *REL* and *MYD88* [13,43–47]. Overall, our approach identified already known regulators but also nominated new candidate genes involved in M1 / M2 polarization of human macrophages.

### Pharmacological validation of selected candidate M2 mediators

**Fig 3:**
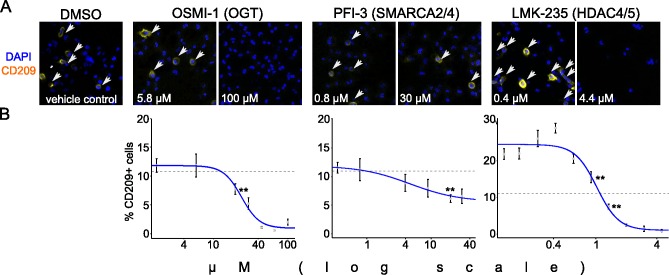
Validation of selected M2 polarization modulators with small molecule inhibitors. (A) Representative images of indirect immunofluorescence staining for CD209 positive M2 macrophages. Cells were treated with OSMI-1, PFI-3 and LMK-235 targeting OGT, SMARCA2/4 and HDAC4/5, respectively, and compared to DMSO control. From the treatments a lower and the highest concentrations are shown. The representative images shown were processed with Fiji and CD209 signal was pseudo-colored yellow. (B) Dose response curves representing percent of CD209 positive cells at different concentrations of compound treatment. Values from 4 independent wells for each condition were averaged and plotted as data points. Error bars indicate the standard deviation. Dashed horizontal lines mark the average percent (11%) of positive cells in DMSO controls (n = 48 wells; standard deviation = 2.25). **The lowest or highest concentration with a significant effect (unpaired Student’s t-test, p < 0.01) compared to the DMSO control.

Next we strived to validate candidate M2 mediators focusing on genes which scored in more than one contrast, were not identified as essential genes (S1 Fig) and for which tool compounds active in cellular assays have been reported. We converted our flow cytometric assay to an imaging assay to determine the percent of CD209+ cells after M2 polarization (Fig 3A and 3B and Materials and Methods). In this setup the average percent of CD209+ cells was 11%, allowing detection of both inhibitory and enhancing effects. We used OSMI-1, LMK-235 and PFI-3 to inhibit enzymatic activities of OGT, HDAC4/5 or the bromo-domain of SMARC4/5, respectively, using previously reported concentration ranges to achieve cellular activity [48–51]. We used concentrations that correspond to the previously reported ranges to achieve cellular activity for these compounds. We plotted the percentage of CD209+ cells corresponding to various concentrations of compound treatments. At concentrations above 1.32 μM LMK-235 decreased the ratio of CD209+ cells consistent with the shRNA results, but surprisingly we observed the opposite effect at concentrations below 0.9 μM. We did not further explore the minimal concentration at which the enhancing effect would be still observed. The SMARCA2/4 bromo-domain inhibitor PFI-3 had a mild but statistically significant effect at higher concentrations to reduce CD209 expression. OGT inhibitor OSMI-1 showed a statistically significant inhibition of CD209 above 20 μM concentrations. In summary, the compound treatment experiments confirmed the shRNA results for OGT, SMARCA4 and HDAC5.

### OGT modulates the transcription of several M2 polarization genes

To our knowledge previous studies on OGTs function in macrophage biology were limited to M1 activation and did not use loss of function genetic approaches or human cell models for the experiments [52,53]

To confirm the validity of OGT as a modulator of macrophage polarization we carried out validation experiments using orthogonal approaches.

**Fig 4:**
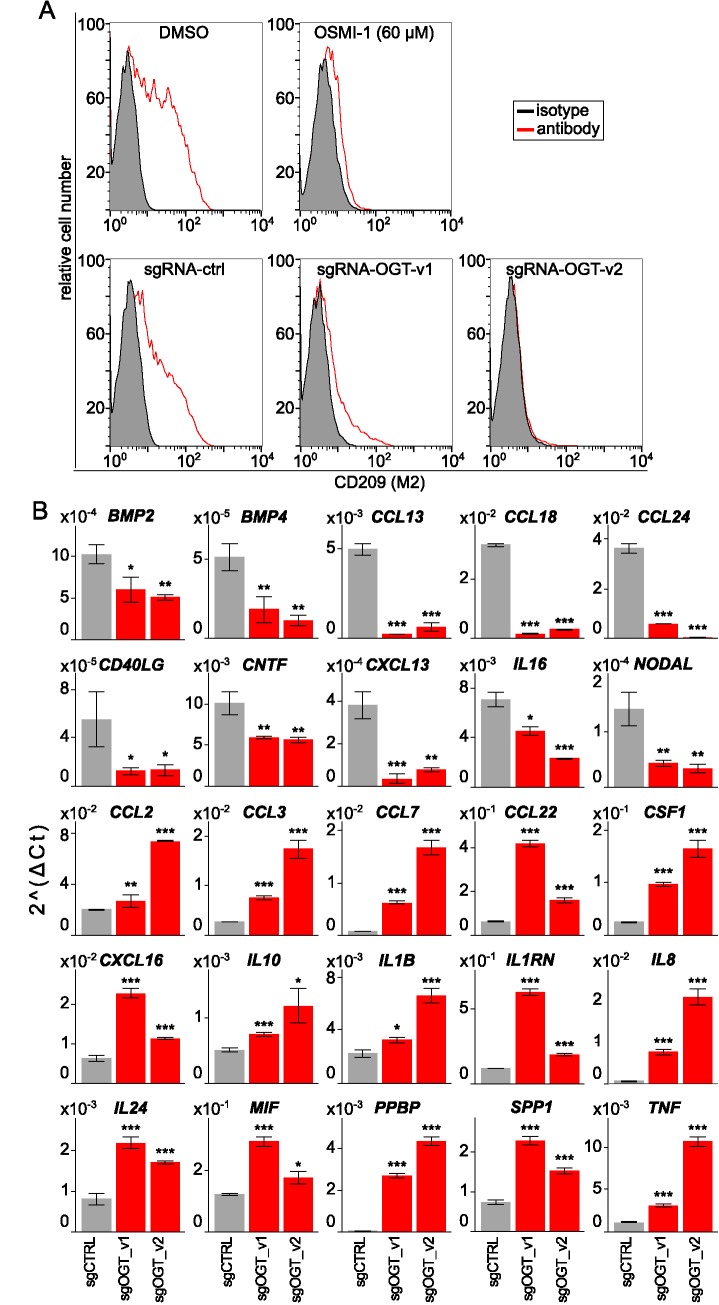
Validation of OGT as a modulator of M2 polarization. (A) Flow cytometric analysis of the M2 (CD209) cell surface marker after inducing M2 polarization in OGT targeted or control cells. OGT was targeted with the small molecule OSMI-1 or CRISPR with two independent sgRNA constructs, and compared to the corresponding DMSO or scrambled controls. (B) The 25 OGT regulated genes identified from 84 probed cytokines and chemokines are shown. mRNA levels were quantified by qPCR on control and OGT targeted THP-1 M2 macrophages (n = 3). Data are presented as mean ± SD; unpaired Student’s t test: *p < 0.05; **p < 0.01, ***p < 0.001.

First the high content assay results were confirmed with flow cytometric analysis of THP-1 macrophages pre-treated with 60 μM OSMI-1 and stimulated with IL4 / IL13 (Fig 4A top panel). DMSO treatment served as control and flow cytometric analysis of CD209 expression was used as readout. In line with the results from the high content readout we observed that pharmacological inhibition of OGT phenocopied the effect of shRNA knock down in THP-1 M2 macrophages.

The shRNA and small molecule experiments validated OGT function in M2 polarization by two methods representing different modes of action. To further strengthen our findings OGT was validated with the CRISPR-Cas9 system. Three sgRNAs targeting *OGT* were tested for their ability to reduce OGT protein level by western blot (S3 Fig). Two sgRNAs against *OGT* showed strong reduction of OGT protein level and these were further evaluated for their effect on macrophage polarization by flow cytometry (Fig 4A, lower panel). In line with the orthogonal approaches, we measured reduced M2 marker (CD209) expression in *OGT* targeted THP-1 after IL4 / IL13 stimulation. The results were comparable to *OGT* knock down with individual shRNAs (S3 Fig). To evaluate whether this effect was specific for the CD209 marker, or the M2 gene expression signature was affected more broadly, we analyzed mRNA expression levels of genes relevant for innate immune response (Fig 4B and S4 Table). The analysis revealed that depletion of OGT had negative effect on M2-specific gene expression (*CCL13*, *CCL18* and *CCL24*) in response to IL4 / IL13, whereas M1-specific genes such as *CCL2*, *CCL3*, *IL1B* and *TNF* were upregulated. Interestingly, three M2 subtype associated markers (*CCL22*, *CXCL16* and *IL10*) were upregulated in the targeted cells. Taken together, these results establish OGT as a mediator of IL4 / IL13 induced M2 polarization in human macrophages.

## Discussion

The advents of pooled lentiviral shRNA and CRISPR libraries have enabled large scale systematic genetic screens to assess stable loss of function phenotypes in mammalian cells [23–25,27–29]. The possibility to interfere with gene function for an extended period is particularly important to study epigenetic regulation which may require prolonged perturbation to trigger changes in chromatin states. However, lack of efficient gene transfer or availability of certain cell types may limit the use of these powerful approaches. Human primary macrophages represent such an example, where systematic identification of regulators of M1 and M2 polarization could significantly contribute to our understanding of host immune response, inflammation and maintenance of tissue homeostasis. To overcome the above-mentioned limitations we used the human THP-1 model allowing cell autonomous readouts of innate immune activation and we devised multidimensional genetic screens addressing human M1 / M2 polarization phenomenon.

The application of appropriate macrophage activation stimuli ensured gene expression patterns which are characteristic of the respective macrophage subtype function (Fig 1). It should be noted that there can be significant differences in the response of polarization markers depending on the species (mouse vs. human), the applied stimuli and source of cells [31,32,39,54–56]. These should be considered when extrapolating from these results. According to our own data as well as published reports, the THP-1 model recapitulated key aspects of human macrophage polarization when compared to respective primary cells [32,40] (Fig 1). Importantly, we observed similar gene expression changes of well-established markers for both M1 and M2 phenotypes in THP-1 and human primary macrophages [32,40,42] (Fig 1E). Likewise, the cytokine / chemokine repertoires of THP-1 macrophages were similar to corresponding primary human macrophage subtypes (Fig 1D). Collectively, THP-1 cells serve as a well suited model system to study human-related aspects of macrophage polarization, with some limitations deriving from its leukemic cell line nature [32].

Notably, M1 and M2 polarized THP-1 were positive for CD38 and CD209, respectively, thus allowing sorting of specific macrophage phenotypes from the pool of cells. This was further exploited in the subsequent screening experiments with a pooled shRNA library yielding identification of novel regulators of the M1 and M2 phenotypes (Fig 2). In M1 / M2 polarization screens we collected five phenotypically and functionally distinct fractions, thus allowing extensive analysis of pro- and anti-inflammatory responses. Importantly, use of a 20 shRNA deep pooled lentiviral library ensured robust statistical power to call hits from the 648 genes targeted. The applied analysis pipeline compared all sorted macrophage fractions in different screening modes (suppressor and mediator) giving rise to 10 different analysis modules (Fig 2, Table 1 and S3 Table). The RSA method ranks genes but an arbitrary cutoff to call hits is necessary. We prioritized the top 10 (1.5% of genes in our experiments) hits from each analysis as a pragmatic compromise between sensitivity and confidence in calling hits. Since we had 10 different analysis modules, the hits could be further prioritized based on the number of comparisons they scored in as mediator or suppressor (Table 1 and S3 Table). Importantly, the pattern in which a given gene scored in the different comparisons may reflect functional differences. Indeed, the data suggested OGT to be a mediator of M2 polarization, but also a suppressor of the M1 phenotype, indicating involvement in regulating macrophage polarization. On the contrary, MYC scored as a suppressor of both M1 and M2, which may reflect its function in promoting cell cycle progression while counteracting differentiation programs.

To demonstrate the value of our approach in discovering regulators of human macrophage biology with yet unappreciated functions we focused the validation experiments on M2 regulation by OGT, HDAC5 and SMARCA4 (Figs 3 and 4A). We used high content imaging to quantify the percent of M2 (CD209+) cells in response to pharmacological inhibition with OSMI-1 (OGT), LMK-235 (HDAC4/5) and PFI-3 (SMARCA2/4 bromo-domain) [48–51]. Applying these small molecule inhibitors confirmed the shRNA results, and supports the hypothesis that these factors would be required for M2 polarization. Surprisingly, lower concentrations of LMK-235 increased CD209 expression. This could reflect differences in sensitivity of various loci to HDAC5 inhibition resulting in diverging concentration dependent outcomes. Since LMK-235 also inhibits HDAC4, we cannot exclude the possibility that this effect is independent of HDAC5 [51]. However, only HDAC5 scored in our shRNA screen as an M2 mediator, and HDAC4 was not detected as an M2 suppressor arguing for HDAC5 specific effects. In addition HDAC5 has been linked to the inflammatory response of macrophages by a previous study, supporting the idea that it has a multi-facetted role in regulating polarization [57]. The SMARCA2/4 bromo-domain inhibitor PFI-3 showed partial but statistically significant inhibition of CD209. Importantly, PFI-3 inhibition does not affect the enzymatic activity of SMARCA2/4 and the bromo-domain seems dispensable for SMARCA2/4 function in previously reported proliferation assays including THP-1 monocytes [49]. Thus, our observation that PFI-3 modulates the M2 phenotype in THP-1 could indicate that SMARCA4 bromo-domain function is more relevant in post mitotic cells. Interestingly, a phosphoproteomic study linked SMARCA4 to macrophage multinucleation and testing if the two phenomena are linked could be an interesting question for future studies [58]. Finally, since PFI-3 also inhibits the bromo-domain of SMARCA2, we cannot exclude the possibility that this effect is independent of SMARCA4 [49]. However, *SMARCA2* did not score in our shRNA screens arguing for SMARCA4 specific effects. Additionally, OGT was also validated in the high content assay with the small molecule inhibitor OSMI-1, which was further confirmed in flow cytometry experiments (Figs 3 and 4A) [48]. Both pharmacological inhibition and sgRNA targeting of OGT phenocopied the inhibitory effect on M2 polarization observed with shRNA knock down (Fig 4A). Taken together, three different approaches (shRNA, small molecule inhibition and CRISPR) consistently validated *OGT* as a gene required for IL4 / IL13 induced M2 polarization in THP-1 macrophages.

As part of our validation experiments for *OGT* we measured the mRNA levels of a subset of innate immune response relevant genes (Fig 4B). This approach did not interrogate the whole transcriptome, but still allowed testing if *OGT* controls a broader set of M2 relevant genes or only a subset. The expression of several macrophage subtype markers changed consistently with the proposed function of OGT to mediate M2 polarization [31]. We also noted three markers linked to the M2 state (*CCL22*, *CXCL16* and *IL10*) that increased expression upon *OGT* targeting. Interestingly, a recent study showed that siRNA inhibition of *Ogt* resulted in increased IL10 expression in mouse intestinal macrophages which would be in line with our results [53]. Another report suggested that *Ogt* dampened M1 activation in mouse RAW264.7 macrophages involving NF-κB, Sin3a and Ep300 [52]. Effects on M2 polarization were not investigated and loss of function genetic evidence was not shown in that study. These results suggest, as expected, that OGT controls parts of the response to IL4 / IL13 in THP-1 cells and orthogonal pathways obviously exist that account for the full M2 polarization program. These gene specific effects could be mediated by locus specific recruitment of OGT to chromatin and by the O-GlcNAcylation of various target proteins. Indeed, OGT fulfills its multiple roles as a key regulator of signaling and transcription as well as an integrator of metabolic signaling *via* numerous interaction partners [59]. Based on our results with small molecule OGT inhibition we hypothesize that the enzymatic activity of OGT is required to exert its function as an M2 regulator (Figs 3 and 4A). Our results add to the understanding of OGT function in macrophages and suggest an important role in regulating M2 polarization in humans.

Collectively, we provide an effective approach for large scale genetic screens uncovering genes involved in human macrophage polarization. The use of a lentiviral library combined with a phenotypic readout allowed investigating complex phenotypes requiring extended time to develop. Our approach can be easily scaled up using genome-wide CRISPR / shRNA libraries and could potentially decipher near-complete pathways relevant for macrophage biology in humans.

## Supporting information

S1 Fig Phenotypic pooled shRNA screen to identify essential genes. (A) Strategy to identify genes knock-down of which facilitates cell death. (B) Identified essential genes.
(EPS)

S2 Fig Reproducibility of the shRNA barcode counts in replicas. (EPS)

S3 Fig Validation of individual shRNA and sgRNA constructs. (A) Flow cytometric analysis of the M2 (CD209) cell surface marker in M2 polarization induced THP-1 cells after transduction with lentiviral shRNA constructs targeting OGT or luciferase-control. Individual constructs correspond to the top 3 shRNAs from the pooled library targeting OGT. (B) Western blot detecting OGT protein in THP-1 Mo stably expressing Cas9 without sgRNA, or transduced with control (CTRL), or OGT targeting sgRNAs, respectively. ACTB was used as a loading control.
(EPS)

S1 Table Single cell intensity values from imaging experiments. (XLSX)

S2 Table shRNA counts. (XLSX)

S3 Table Overview of the top 10 hits in the different contrasts and modes. (XLSX)

S4 Table qPCR Ct values. (XLSX)

## References

[1] Geissmann F, Manz MG, Jung S, Sieweke MH, Merad M, Ley K. Development of monocytes, macrophages, and dendritic cells. Science. 2010;327: 656–61. doi: 10.1126/science.1178331 20133564 10.1126/science.1178331 PMC2887389

[2] Gordon S, Taylor PR. Monocyte and macrophage heterogeneity. Nat Rev Immunol. 2005;5: 953–64. doi: 10.1038/nri1733 16322748 10.1038/nri1733

[3] Mantovani A, Biswas SK, Galdiero MR, Sica A, Locati M. Macrophage plasticity and polarization in tissue repair and remodelling. Journal of Pathology. 2013 pp. 176–185. doi: 10.1002/path.4133 23096265 10.1002/path.4133

[4] Sica A, Mantovani A. Macrophage plasticity and polarization: In vivo veritas. Journal of Clinical Investigation. 2012 pp. 787–795. doi: 10.1172/JCI59643 22378047 10.1172/JCI59643 PMC3287223

[5] Biswas SK, Mantovani A. Macrophage plasticity and interaction with lymphocyte subsets: cancer as a paradigm. Nat Immunol. 2010;11: 889–896. doi: 10.1038/ni.1937 20856220 10.1038/ni.1937

[6] Gautier EL, Shay T, Miller J, Greter M, Jakubzick C, Ivanov S, et al Gene-expression profiles and transcriptional regulatory pathways that underlie the identity and diversity of mouse tissue macrophages. Nat Immunol. 2012;13: 1118–28. doi: 10.1038/ni.2419 23023392 10.1038/ni.2419 PMC3558276

[7] Hagemann T, Lawrence T, McNeish I, Charles K a, Kulbe H, Thompson RG, et al “Re-educating” tumor-associated macrophages by targeting NF-kappaB. J Exp Med. 2008;205: 1261–1268. doi: 10.1084/jem.20080108 18490490 10.1084/jem.20080108 PMC2413024

[8] Mylonas KJ, Nair MG, Prieto-Lafuente L, Paape D, Allen JE. Alternatively activated macrophages elicited by helminth infection can be reprogrammed to enable microbial killing. J Immunol. 2009;182: 3084–3094. doi: 10.4049/jimmunol.0803463 19234205 10.4049/jimmunol.0803463

[9] Stout RD, Jiang C, Matta B, Tietzel I, Watkins SK, Suttles J. Macrophages sequentially change their functional phenotype in response to changes in microenvironmental influences. J Immunol. 2005;175: 342–349. doi: 10.4049/jimmunol.175.1.342 15972667 10.4049/jimmunol.175.1.342

[10] Gordon S. Alternative activation of macrophages. Nat Rev Immunol. 2003;3: 23–35. doi: 10.1038/nri978 12511873 10.1038/nri978

[11] Murray PJ, Wynn TA. Protective and pathogenic functions of macrophage subsets. Nat Rev Immunol. 2011;11: 723–737 doi: 10.1038/Nri3073 21997792 10.1038/nri3073 PMC3422549

[12] Ivashkiv LB. Epigenetic regulation of macrophage polarization and function. Trends in Immunology. 2013 pp. 216–223. doi: 10.1016/j.it.2012.11.001 23218730 10.1016/j.it.2012.11.001 PMC3647003

[13] Belkina AC, Nikolajczyk BS, Denis G V. BET protein function is required for inflammation: Brd2 genetic disruption and BET inhibitor JQ1 impair mouse macrophage inflammatory responses. J Immunol. 2013;190: 3670–8. doi: 10.4049/jimmunol.1202838 23420887 10.4049/jimmunol.1202838 PMC3608815

[14] Chen X, Barozzi I, Termanini A, Prosperini E, Recchiuti A, Dalli J, et al Requirement for the histone deacetylase Hdac3 for the inflammatory gene expression program in macrophages. Proc Natl Acad Sci U S A. 2012;109: E2865–74. doi: 10.1073/pnas.1121131109 22802645 10.1073/pnas.1121131109 PMC3479529

[15] De Santa F, Narang V, Yap ZH, Tusi BK, Burgold T, Austenaa L, et al Jmjd3 contributes to the control of gene expression in LPS-activated macrophages. EMBO J. 2009;28: 3341–52. doi: 10.1038/emboj.2009.271 19779457 10.1038/emboj.2009.271 PMC2752025

[16] Ishii M, Wen H, Corsa CAS, Liu T, Coelho AL, Allen RM, et al Epigenetic regulation of the alternatively activated macrophage phenotype. Blood. 2009;114: 3244–3254. doi: 10.1182/blood-2009-04-217620 19567879 10.1182/blood-2009-04-217620 PMC2759649

[17] Nicodeme E, Jeffrey KL, Schaefer U, Beinke S, Dewell S, Chung C, et al Suppression of inflammation by a synthetic histone mimic. Nature. 2010;468: 1119–1123. doi: 10.1038/nature09589 21068722 10.1038/nature09589 PMC5415086

[18] Roger T, Lugrin J, Le Roy D, Goy G, Mombelli M, Koessler T, et al Histone deacetylase inhibitors impair innate immune responses to Toll-like receptor agonists and to infection. Blood. 2011;117: 1205–1217. doi: 10.1182/blood-2010-05-284711 20956800 10.1182/blood-2010-05-284711

[19] Satoh T, Takeuchi O, Vandenbon A, Yasuda K, Tanaka Y, Kumagai Y, et al The Jmjd3-Irf4 axis regulates M2 macrophage polarization and host responses against helminth infection. Nat Immunol. 2010;11: 936–944. doi: 10.1038/ni.1920 20729857 10.1038/ni.1920

[20] Sun J, Katz S, Dutta B, Wang Z, Fraser IDC. Genome-wide siRNA screen of genes regulating the LPS-induced TNF-α response in human macrophages. Sci Data. The Author(s); 2017;4: 170007 doi: 10.1038/sdata.2017.7 28248930 10.1038/sdata.2017.7 PMC5332009

[21] Troegeler A, Lastrucci C, Duval C, Tanne A, Cougoule C, Maridonneau-Parini I, et al An efficient siRNA-mediated gene silencing in primary human monocytes, dendritic cells and macrophages. Immunol Cell Biol. 2014;92: 699–708. doi: 10.1038/icb.2014.39 24890643 10.1038/icb.2014.39

[22] Zhou H, DeLoid G, Browning E, Gregory DJ, Tan F, Bedugnis AS, et al Genome-Wide RNAi Screen in IFN-γ-Treated Human Macrophages Identifies Genes Mediating Resistance to the Intracellular Pathogen Francisella tularensis. Neyrolles O, editor. PLoS One. Public Library of Science; 2012;7: e31752 doi: 10.1371/journal.pone.0031752 22359626 10.1371/journal.pone.0031752 PMC3281001

[23] Hoffman GR, Rahal R, Buxton F, Xiang K, McAllister G, Frias E, et al Functional epigenetics approach identifies BRM/SMARCA2 as a critical synthetic lethal target in BRG1-deficient cancers. Proc Natl Acad Sci. 2014;111: 3128–3133. doi: 10.1073/pnas.1316793111 24520176 10.1073/pnas.1316793111 PMC3939885

[24] Luo B, Cheung HW, Subramanian A, Sharifnia T, Okamoto M, Yang X, et al Highly parallel identification of essential genes in cancer cells. Proc Natl Acad Sci U S A. 2008;105: 20380–5. doi: 10.1073/pnas.0810485105 19091943 10.1073/pnas.0810485105 PMC2629277

[25] Parnas O, Jovanovic M, Eisenhaure TM, Herbst RH, Dixit A, Ye CJ, et al A Genome-wide CRISPR Screen in Primary Immune Cells to Dissect Regulatory Networks. Cell. 2015;162: 675–686. doi: 10.1016/j.cell.2015.06.059 26189680 10.1016/j.cell.2015.06.059 PMC4522370

[26] Su W-C, Chen Y-C, Tseng C-H, Hsu PW-C, Tung K-F, Jeng K-S, et al Pooled RNAi screen identifies ubiquitin ligase Itch as crucial for influenza A virus release from the endosome during virus entry. Proc Natl Acad Sci U S A. 2013;110: 17516–21. doi: 10.1073/pnas.1312374110 24101521 10.1073/pnas.1312374110 PMC3808593

[27] Dejesus R, Moretti F, McAllister G, Wang Z, Bergman P, Liu S, et al Functional CRISPR screening identifies the ufmylation pathway as a regulator of SQSTM1/p62. Elife. 2016;5 doi: 10.7554/eLife.17290 27351204 10.7554/eLife.17290 PMC4924995

[28] Estoppey D, Hewett JW, Guy CT, Harrington E, Thomas JR, Schirle M, et al Identification of a novel NAMPT inhibitor by CRISPR/Cas9 chemogenomic profiling in mammalian cells. Sci Rep. Nature Publishing Group. 2017;7: 42728 doi: 10.1038/srep42728 28205648 10.1038/srep42728 PMC5311948

[29] Mounir Z, Korn JM, Westerling T, Lin F, Kirby CA, Schirle M, et al ERG signaling in prostate cancer is driven through PRMT5-dependent methylation of the androgen receptor. Elife. 2016;5 doi: 10.7554/eLife.13964 27183006 10.7554/eLife.13964 PMC4909395

[30] Li Q, Karim AF, Ding X, Das B, Dobrowolski C, Gibson RM, et al Novel high throughput pooled shRNA screening identifies NQO1 as a potential drug target for host directed therapy for tuberculosis. Sci Rep. Nature Publishing Group; 2016;6: 1–18. doi: 10.1038/srep27566 27297123 10.1038/srep27566 PMC4906352

[31] Murray PJ, Allen JE, Biswas SK, Fisher EA, Gilroy DW, Goerdt S, et al Macrophage Activation and Polarization: Nomenclature and Experimental Guidelines [Internet]. Immunity. Elsevier; 2014 pp. 14–20. doi: 10.1016/j.immuni.2014.06.008 25035950 10.1016/j.immuni.2014.06.008 PMC4123412

[32] Spiller KL, Wrona EA, Romero-Torres S, Pallotta I, Graney PL, Witherel CE, et al Differential gene expression in human, murine, and cell line-derived macrophages upon polarization. Exp Cell Res. 2016;347: 1–13. doi: 10.1016/j.yexcr.2015.10.017 26500109 10.1016/j.yexcr.2015.10.017

[33] Mestas J, Hughes CCW. Of mice and not men: differences between mouse and human immunology. J Immunol. 2004;172: 2731–2738. doi: 10.4049/jimmunol.172.5.2731 14978070 10.4049/jimmunol.172.5.2731

[34] Murray PJ, Wynn T a. Obstacles and opportunities for understanding macrophage polarization. J Leukoc Biol. 2011;89: 557–563. doi: 10.1189/jlb.0710409 21248152 10.1189/jlb.0710409 PMC3058818

[35] König R, Chiang C, Tu BP, Yan SF, DeJesus PD, Romero A, et al A probability-based approach for the analysis of large-scale RNAi screens. Nat Methods. 2007;4: 847–9. doi: 10.1038/nmeth1089 17828270 10.1038/nmeth1089

[36] Livak KJ, Schmittgen TD. Analysis of relative gene expression data using real-time quantitative PCR and the 2^-ΔΔCT Method. Methods. 2001;25: 402–408. doi: 10.1006/meth.2001.1262 11846609 10.1006/meth.2001.1262

[37] Kamentsky L, Jones TR, Fraser A, Bray MA, Logan DJ, Madden KL, et al Improved structure, function and compatibility for cellprofiler: Modular high-throughput image analysis software. Bioinformatics. 2011;27: 1179–1180. doi: 10.1093/bioinformatics/btr095 21349861 10.1093/bioinformatics/btr095 PMC3072555

[38] Schindelin J, Arganda-Carreras I, Frise E, Kaynig V, Longair M, Pietzsch T, et al Fiji: an open-source platform for biological-image analysis. Nat Methods. 2012;9: 676–82. doi: 10.1038/nmeth.2019 22743772 10.1038/nmeth.2019 PMC3855844

[39] Daigneault M, Preston JA, Marriott HM, Whyte MKB, Dockrell DH. The Identification of Markers of Macrophage Differentiation in PMA-Stimulated THP-1 Cells and Monocyte-Derived Macrophages. Doherty TM, editor. PLoS One. Public Library of Science; 2010;5: e8668 doi: 10.1371/journal.pone.0008668 20084270 10.1371/journal.pone.0008668 PMC2800192

[40] Chanput W, Mes JJ, Wichers HJ. THP-1 cell line: An in vitro cell model for immune modulation approach. International Immunopharmacology. 2014 pp. 37–45. doi: 10.1016/j.intimp.2014.08.002 25130606 10.1016/j.intimp.2014.08.002

[41] Martinez FO, Helming L, Gordon S. Alternative Activation of Macrophages: An Immunologic Functional Perspective. Annu Rev Immunol. 2009;27: 451–483. doi: 10.1146/annurev.immunol.021908.132532 19105661 10.1146/annurev.immunol.021908.132532

[42] Martinez FO, Gordon S. The M1 and M2 paradigm of macrophage activation: time for reassessment. F1000Prime Rep. 2014;6: 13 doi: 10.12703/P6-13 24669294 10.12703/P6-13 PMC3944738

[43] Courtine E, Cagnard N, Mazzolini J, Antona M, Pène F, Fitting C, et al Combined loss of cRel/p50 subunits of NF-κB leads to impaired innate host response in sepsis. Innate Immun. 2012;18: 753–63. doi: 10.1177/1753425912440296 22408080 10.1177/1753425912440296

[44] Fullard N, Wilson CL, Oakley F. Roles of c-Rel signalling in inflammation and disease. International Journal of Biochemistry and Cell Biology. 2012 pp. 851–860. doi: 10.1016/j.biocel.2012.02.017 22405852 10.1016/j.biocel.2012.02.017

[45] Pittet LA, Quinton LJ, Yamamoto K, Robson BE, Ferrari JD, Algül H, et al Earliest innate immune responses require macrophage RelA during pneumococcal pneumonia. Am J Respir Cell Mol Biol. 2011;45: 573–581. doi: 10.1165/rcmb.2010-0210OC 21216972 10.1165/rcmb.2010-0210OC PMC3175578

[46] Shi S, Nathan C, Schnappinger D, Drenkow J, Fuortes M, Block E, et al MyD88 primes macrophages for full-scale activation by interferon-gamma yet mediates few responses to Mycobacterium tuberculosis. J Exp Med. 2003;198: 987–97. doi: 10.1084/jem.20030603 14517275 10.1084/jem.20030603 PMC2194223

[47] Courtine E, Pène F, Cagnard N, Toubiana J, Fitting C, Brocheton J, et al Critical role of cRel subunit of NF-κB in sepsis survival. Infect Immun. 2011;79: 1848–1854. doi: 10.1128/IAI.00021-11 21343350 10.1128/IAI.00021-11 PMC3088128

[48] Ortiz-Meoz RF, Jiang J, Lazarus MB, Orman M, Janetzko J, Fan C, et al A Small Molecule That Inhibits OGT Activity in Cells. ACS Chem Biol. 2015;10: 1392–1397. doi: 10.1021/acschembio.5b00004 25751766 10.1021/acschembio.5b00004 PMC4475500

[49] Vangamudi B, Paul TA, Shah PK, Kost-Alimova M, Nottebaum L, Shi X, et al The SMARCA2/4 ATPase domain surpasses the bromodomain as a drug target in SWI/SNF-mutant cancers: Insights from cDNA rescue and PFI-3 inhibitor studies. Cancer Res. 2015;75: 3865–3878. doi: 10.1158/0008-5472.CAN-14-3798 26139243 10.1158/0008-5472.CAN-14-3798 PMC4755107

[50] Li A, Liu Z, Li M, Zhou S, Xu Y, Xiao Y, et al HDAC5, a potential therapeutic target and prognostic biomarker, promotes proliferation, invasion and migration in human breast cancer. Oncotarget. 2016;7: 37966–37978. doi: 10.18632/oncotarget.9274 27177225 10.18632/oncotarget.9274 PMC5122364

[51] Marek L, Hamacher A, Hansen FK, Kuna K, Gohlke H, Kassack MU, et al Histone deacetylase (HDAC) inhibitors with a novel connecting unit linker region reveal a selectivity profile for HDAC4 and HDAC5 with improved activity against chemoresistant cancer cells. J Med Chem. 2013;56: 427–436. doi: 10.1021/jm301254q 23252603 10.1021/jm301254q

[52] Hwang SY, Hwang JS, Kim SY, Han IO. O-GlcNAc transferase inhibits LPS-mediated expression of inducible nitric oxide synthase through an increased interaction with mSin3A in RAW264.7 cells. Am J Physiol Cell Physiol. 2013;305: C601–8. doi: 10.1152/ajpcell.00042.2013 23824843 10.1152/ajpcell.00042.2013

[53] Li X, Zhang Z, Li L, Gong W, Lazenby AJ, Swanson BJ, et al Myeloid-derived cullin 3 promotes STAT3 phosphorylation by inhibiting OGT expression and protects against intestinal inflammation. J Exp Med. 2017;214: jem.20161105. doi: 10.1084/jem.20161105 28280036 10.1084/jem.20161105 PMC5379975

[54] Italiani P, Mazza EMC, Lucchesi D, Cifola I, Gemelli C, Grande A, et al Transcriptomic profiling of the development of the inflammatory response in human monocytes in vitro. PLoS One. Public Library of Science; 2014;9: e87680 doi: 10.1371/journal.pone.0087680 24498352 10.1371/journal.pone.0087680 PMC3912012

[55] Beyer M, Mallmann MR, Xue J, Staratschek-Jox A, Vorholt D, Krebs W, et al High-Resolution Transcriptome of Human Macrophages. Zirlik A, editor. PLoS One. Public Library of Science; 2012;7: e45466 doi: 10.1371/journal.pone.0045466 23029029 10.1371/journal.pone.0045466 PMC3448669

[56] Jablonski KA, Amici SA, Webb LM, Ruiz-Rosado J de D, Popovich PG, Partida-Sanchez S, et al Novel Markers to Delineate Murine M1 and M2 Macrophages. Olszewski MA, editor. PLoS One. Public Library of Science; 2015;10: e0145342 doi: 10.1371/journal.pone.0145342 26699615 10.1371/journal.pone.0145342 PMC4689374

[57] Poralla L, Stroh T, Erben U, Sittig M, Liebig S, Siegmund B, et al Histone deacetylase 5 regulates the inflammatory response of macrophages. J Cell Mol Med. Wiley-Blackwell; 2015;19: 2162–2171. doi: 10.1111/jcmm.12595 26059794 10.1111/jcmm.12595 PMC4568921

[58] Rotival M, Ko J-H, Srivastava PK, Kerloc’h A, Montoya A, Mauro C, et al Integrating Phosphoproteome and Transcriptome Reveals New Determinants of Macrophage Multinucleation. Mol Cell Proteomics. American Society for Biochemistry and Molecular Biology; 2015;14: 484–498. doi: 10.1074/mcp.M114.043836 25532521 10.1074/mcp.M114.043836 PMC4349971

[59] Lewis BA, Hanover JA. O-GlcNAc and the epigenetic regulation of gene expression [Internet]. Journal of Biological Chemistry. in Press; 2014 pp. 34440–34448. doi: 10.1074/jbc.R114.595439 25336654 10.1074/jbc.R114.595439 PMC4263851

